# Reassessing granzyme B: unveiling perforin-independent versatility in immune responses and therapeutic potentials

**DOI:** 10.3389/fimmu.2024.1392535

**Published:** 2024-05-23

**Authors:** Raylynn Thompson, Xuefang Cao

**Affiliations:** ^1^ Marlene and Stewart Greenebaum Comprehensive Cancer Center, University of Maryland Baltimore School of Medicine, Baltimore, MD, United States; ^2^ Department of Microbiology and Immunology, University of Maryland Baltimore School of Medicine, Baltimore, MD, United States

**Keywords:** granzyme B, perforin, cytotoxic lymphocytes, immune response, inflammatory disorders

## Abstract

The pivotal role of Granzyme B (GzmB) in immune responses, initially tied to cytotoxic T lymphocytes (CTLs) and natural killer (NK) cells, has extended across diverse cell types and disease models. A number of studies have challenged conventional notions, revealing GzmB activity beyond apoptosis, impacting autoimmune diseases, inflammatory disorders, cancer, and neurotoxicity. Notably, the diverse functions of GzmB unfold through Perforin-dependent and Perforin-independent mechanisms, offering clinical implications and therapeutic insights. This review underscores the multifaceted roles of GzmB, spanning immunological and pathological contexts, which call for further investigations to pave the way for innovative targeted therapies.

## 1 Introduction

Human and murine Granzymes play important roles in immune responses, crucial for eliminating aberrant cells. GzmB, a serine protease initially identified in the granules of cytotoxic lymphocytes, aids in eliminating infected or transformed cells via inducing apoptotic cell death (1). Subsequent studies further established a critical role of GzmB in regulating immune response via cytotoxic activity on T lymphocytes and antigen presenting cells (2–4). However, another study challenged the conventional understanding of lymphocyte-mediated cytotoxicity by showcasing that *in vivo* cytotoxicity by cytotoxic T lymphocytes (CTLs) remains efficient in the absence of Granzymes A and B, prompting a reevaluation of their roles in cell death and other processes in immune responses (5). Additionally, recent studies have expanded GzmB expression and function to a broader spectrum of other cell types including mast cells, plasmacytoid dendritic cells (pDCs), and B cells (6). For example, the study by Pardo et al. provided significant evidence of GzmB function independent of Perforin in mouse mast cells, expanding its role beyond traditional cytotoxic lymphocytes (7). In inflammatory disorders such as atherosclerosis, mast cell-derived GzmB may contribute to arterial damage; in autoimmune diseases such as Rheumatoid Arthritis (RA), Perforin-independent extracellular GzmB activity may also contribute to tissue destruction (8). Other studies highlighted GzmB expression in pDCs and B cells with regulatory phenotypes (9, 10), which then utilize GzmB to negatively modulate T cell activity. Furthermore, GzmB’s neurotoxic effects appear to transcend T cell-delivered intracellular cytotoxic mechanism in neurological diseases such as Multiple Sclerosis (MS), wherein GzmB was shown to induce neurotoxicity through interacting with the membrane bound receptors (11, 12).

As exemplified by these studies, GzmB, initially known for its role in cytotoxic lymphocyte-mediated immune surveillance, has now extended its influence across diverse contexts. These findings underline GzmB’s roles across multiple diseases, positioning it as a significant entity with clinical relevance and therapeutic potential.

## 2 Reassessing perforin-independent versatility of GzmB in immune responses

Natural Killer (NK) and CTLs are crucial to immune defense, cancer surveillance, and precision medicine. Both cells can eliminate infected or cancerous cells through cytotoxic molecules, including GzmB, initially thought to rely on Perforin for target cell entry (1). However, recent discoveries have expanded GzmB activity to many other cell types and revealed its Perforin-independent actions, broadening its impact in cytotoxic activity as well as non-cytotoxic inflammatory processes. These findings have added complexities to cytotoxic mechanisms, calling for further investigation into the partnering versus separating relationships between GzmB and Perforin in immune defense as well as inflammatory disorders.

### 2.1 GzmB expression in cytotoxic lymphocytes versus other types of immune cells

The understanding of GzmB expression, from transcriptional activation to post-translational modifications, remains a dynamic area of research. In a study conducted in 2005, the direct impact of IL-2 on Perforin and GzmB expression in lymphocytes was revealed, enhancing cytotoxic activity without affecting survival or division of the lymphocyte itself (13). Aside from cytotoxic lymphocytes, a study in 2009 observed that activated human pDCs secrete GzmB, triggered by IL-3 and IL-10, causing suppression of T cell proliferation (9). Additionally, B cell receptor activation along with IL-21 treatment induced proliferation of human B cells with high levels of GzmB expression (10). Furthermore, Pardo et al. presented evidence that skin-, but not lung-associated primary mast cells and *in vitro*-differentiated bone marrow-derived mast cells (BMMC) express GzmB (but not GzmA or Perforin), which is associated with cytoplasmic granules in BMMC and is secreted after Fcϵ-receptor-mediated activation (7). In the meantime, post-translational modifications, such as phosphorylation and proteolytic cleavage, are involved in converting inactive GzmB into its functional form, and may also influence apoptosis induction and inflammatory response. There are ongoing investigations to elucidate the diverse signals that drive GzmB production by different cell types, from gene expression to functional activation, and the respective contributions to immune response and inflammatory condition.

### 2.2 GzmB release: granule exocytosis

Activated lymphocytes including CTLs and NK cells are known to express Perforin and Granzymes, and package these molecules into the cytotoxic granules that are derived from lysosome compartments (14). When a CTL recognizes and binds a target cell, the formation of immunological synapse following MHC-TCR interaction activates linker for activation of T cells (LAT) signalosome, which triggers calcium influx through phospholipase C (PLC) pathway (15, 16). Subsequently, the elevated calcium mobilizes cytotoxic granules to polarize towards the immunological synapse, then Granzymes and Perforin are released through degranulation (17). Interestingly, recent studies have shown that other types of immune cells including B cells, pDCs and mast cells may express and secrete GzmB in the absence of Perforin. However, the molecular machinery governing GzmB release from these conventionally known non-cytotoxic immune cells is not well studied and demands further investigation.

### 2.3 GzmB delivery: dual mechanisms

**Figure 1:**
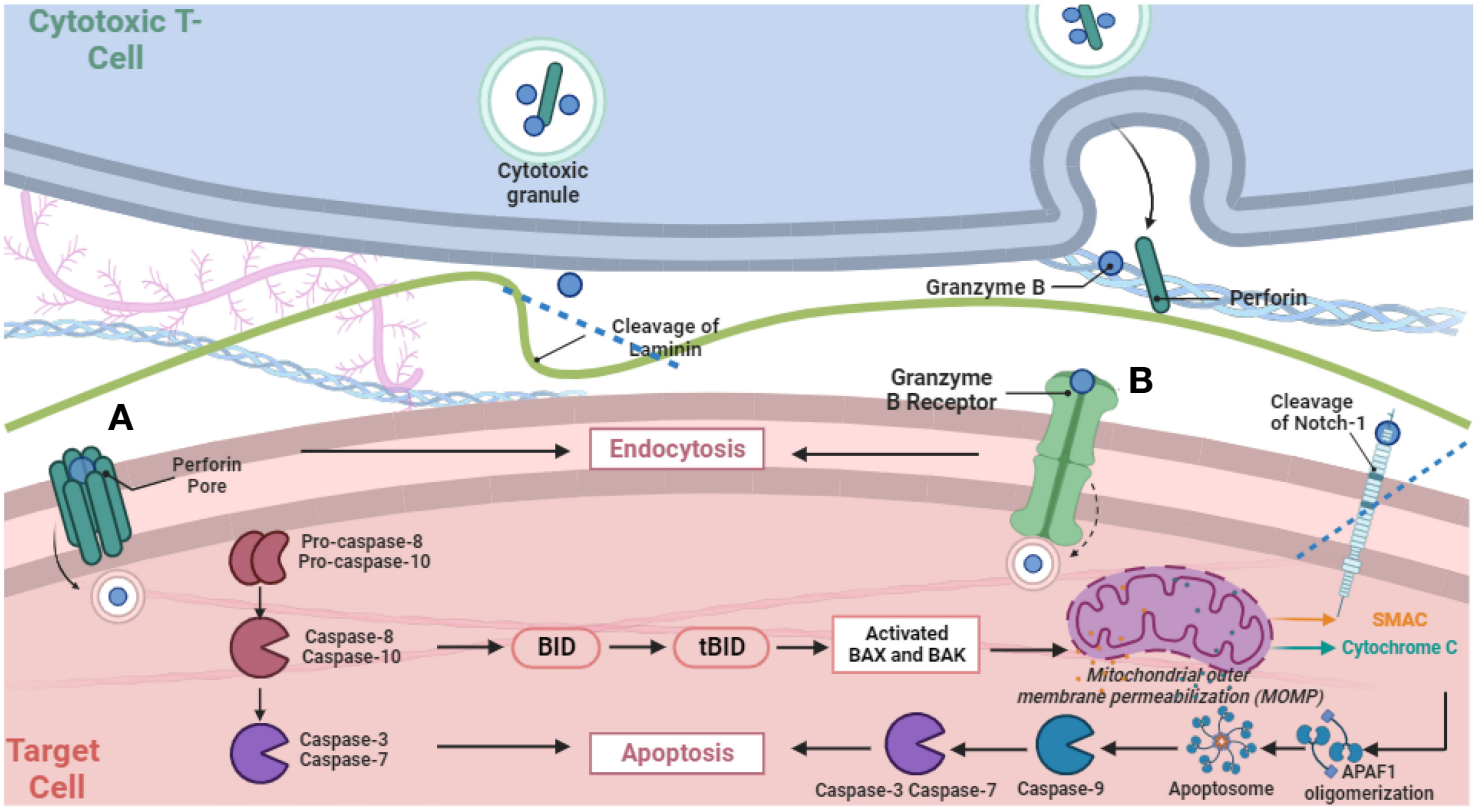
Schematic Representation of GzmB release via degranulation from cytotoxic T cells and subsequently two distinct pathways for delivery into target cells. The figure illustrates pore formation via Perforin polymerization **(A)** and receptor-mediated endocytosis **(B)**, and subsequent intracellular proteolytic activities that activate the cascade of apoptotic cell death.

After release, the dual mechanisms of GzmB delivery—receptor-mediated and Perforin-mediated pathways—underlie its versatile functions (**Figure 1**). The receptor-mediated pathway engages cell surface receptors and involves receptor-mediated endocytosis, impacting protein cleavage and caspase interplay. Meanwhile, the Perforin-dependent route involves extracellular release via the immunological synapse, translocating into target cells to induce apoptosis via caspase cleavage. A recent study exemplified the separation and collaboration between GzmB and Perforin in this process, by examining intracellular damage in intestinal epithelial cells induced by anti-CD3 mAb-activated intra-epithelial lymphocytes (18). It revealed GzmB-dependent yet Perforin-independent DNA fragmentation in intestinal epithelial cells, while GzmB can still concurrently engage Perforin-dependent pathways. This challenges the binary perception of GzmB delivery, emphasizing its nuanced interplay with both mechanisms. Such adaptability adds complexity to immune surveillance, requiring comprehensive investigation into its diverse actions. The relationship between GzmB, Perforin, heparan sulfate, and mannose-6-phosphate receptor (M6PR) underscores their joint role in efficient GzmB delivery. While receptors significantly aid entry, alternative routes highlight GzmB’s versatility. Granule-mediated killing is augmented by heparan sulfate and M6PR, broadening the scope of GzmB’s action in immune responses (19).

#### 2.3.1 Perforin-mediated delivery

The long-held doctrine is that GzmB works hand-in-hand with Perforin to trigger cell death. In the canonical model, GzmB is delivered into target cells through the immunological synapse in a Perforin-dependent manner upon the cell-cell contact between lymphocytes and target cells, followed by cleaving intracellular substrates to execute apoptotic cell death (1). During Perforin-mediated delivery into target cells, GzmB leakage is mitigated through a diffusion-proof immunological synapse that is formed in an MHC-restricted manner. Perforin polymerization within the target cell membrane is believed to form pores that breach the target cell membrane, through which GzmB then translocates into the target cells, initiating intracellular apoptotic pathways (1).

#### 2.3.2 Receptor-mediated endocytosis

Understanding GzmB’s diverse functionalities and its autonomy from Perforin has been a journey, beginning in 1997, which marked a significant revelation in GzmB function independent of Perforin. Exploring the role of cytotoxic cells, specifically lymphokine-activated killer (LAK) cells, against various viral infections, an *in vitro* model showcased LAK cells’ ability to degrade RNA in VSV-infected cells, curtailing infectious virion production. The study demonstrated that GzmB reduced viral transcript levels and induced rapid DNA strand breakage within infected cells, affirming its Perforin-independent efficacy in curbing VSV production (20). Further revelations continued to unravel GzmB’s Perforin-independent activities. In 2000, the focus shifted to receptor-mediated endocytosis, uncovering the cation-independent mannose-6-phosphate receptor (CI-M6PR) as GzmB’s primary binding and uptake receptor (21). It was initially believed that endocytosis and GzmB accumulation within endosomal vesicles, assisted by CI-M6PR, were inadequate for GzmB’s cytosolic delivery. However, subsequent studies revealed GzmB’s independent cytotoxic effects without Perforin. Non-glycosylated GzmB demonstrated selective and potent cytotoxicity to target cells, suggesting potential application in cytotoxic immunoconjugates for targeted cell killing (22). A 2004 study further challenged existing beliefs by demonstrating GzmB’s preference for receptor-mediated endocytosis over pore formation by Perforin. It identified dynamin as an entry receptor on target cells, elucidating its role in GzmB-mediated apoptosis (23). Furthermore, a 2005 study delved into CD4+CD25+ regulatory T cells’ suppression mechanism, unveiling GzmB’s critical role in regulating immune responses. Reducing GzmB levels hindered Treg cells’ efficacy in suppressing other immune cells during direct interactions, challenging the belief that GzmB required additional partners for function (24).

In addition to these two distinct pathways, GzmB was shown to undergo electrostatic exchange from serglycin, another component in cytotoxic granules, which facilitates GzmB delivery to target cells (25). The electrostatic exchange phenomenon may be generalized to multiple uptake pathways, ensuring granzyme influence on a wide variety of target cell types.

## 3 Three locations of function: intracellular, cell surface, extracellular

### 3.1 Intracellular functions: cytotoxic versus non-cytotoxic

In the classical model, intracellular GzmB is detected within cytotoxic lymphocytes or their corresponding target cells following Perforin-mediated delivery (26). Once delivered into target cells, GzmB activates a precise apoptotic cascade via caspase cleavage, notably pro-caspase-8 and pro-caspase-10. This orchestrated process involves a pivotal interaction with Bid, generating tBid and triggering mitochondrial release of cytochrome c. Cytochrome c, along with other factors, activates caspase-9, initiating downstream effector caspases, ultimately causing DNA fragmentation, a defining feature of programmed cell death (27). In contrast, a recent study identified a previously unexpected role of GzmB in triggering sublethal DNA damage response in cancer cells independent of Perforin, where GzmB performs proteolytic function without causing cell death by cleaving intracellular proteins, including ICAD, caspase-7, caspase-8, DDX21, topoisomerase-IIα, and NuMA, indicative of GzmB’s protease activity within cells (28). Interestingly, these intracellular enzymatic activities lead to a non-cytotoxic DNA damage response. Consequently, interferon regulatory transcription factor 3 (IRF3) was activated, which may regulate the expression of a set of interferon-stimulated genes involved in inflammatory response (28).

### 3.2 Cell surface protein and membrane bound receptors

A number of studies have extended Perforin-independent GzmB activity to the membrane surface of target cells. First, NK cell-derived GzmB was reported to specifically bind cell surface-bound heat shock protein 70 (Hsp70) in a process that mediates Perforin-independent GzmB uptake (29). This interaction increases the sensitivity of tumor cells to NK cell-mediated killing. The study identified a 14 amino acid sequence from Hsp70, termed TKD, sensitizing tumor cells to NK cytotoxic activity, orchestrating GzmB release by activated NK cells and inducing apoptosis in Hsp70 membrane-positive tumor cells. These findings support an immunotherapeutic strategy that uses GzmB as a lever to target membrane Hsp70-expressing tumors (30). In addition, in a model of T cell-mediated neuroinflammatory disorder, GzmB was found to mediate neurotoxicity through proteolytic activity on a G-protein-coupled receptor (11). GzmB cleaves and activates the protease-activated receptor-1 (PAR-1) on neuronal cell membrane, leading to increased expression and translocation of the voltage gated potassium channel, Kv1.3 to the neuronal cell membrane, followed by activation of Notch-1 resulting in neurotoxicity (12). These observations suggest that GzmB released from T cells induced neuronal injury and neurite atrophy by interacting with the membrane bound Gi-coupled PAR-1 receptor and subsequently activated Kv1.3 and Notch-1, which was required for GzmB-induced neurotoxicity (12). Furthermore, GzmB displayed Perforin-independent function by cleaving the extracellular domain of Notch-1, yet GzmB cleavage of Notch-1 can occur in all subcellular compartments, during maturation of the Notch-1 receptor, at the membrane, and in the nucleus (31). Cleavage of Notch-1 by GzmB resulted in a loss of transcriptional activity, independent of Notch-1 activation, which may influence target cell proliferation and survival (31).

### 3.3 Extracellular functions

Understanding GzmB’s extracellular functions remains an active research area despite significant progress in deciphering its intracellular mechanisms. Recent studies accentuate the diverse extracellular roles of GzmB, expanding beyond apoptosis, demonstrating its involvement in extracellular matrix (ECM) remodeling and immune regulation (8, 18). Released independently of perforin or GzmA, mast cell-derived GzmB plays a role in ECM modulation, impacting cellular adhesion and migration (27). Also, immunological synapse leakage of GzmB may influence cellular behavior. Anoikis induction in smooth muscle and endothelial cells reveals GzmB’s role in cellular motility and its potential anti-carcinogenic impact. Several ECM substrates for GzmB have been identified, including aggrecan, fibronectin, vitronectin, and laminin. GzmB cleaves vitronectin at the RGD domain, crucial for integrin binding, disrupting cellular-vitronectin interactions and affecting cell adhesion and migration (27). Notably, a recent study using 2-photon microscopy shows that extracellular GzmB activity is important for the migration of primed CD8+ CTLs (32). Upon engagement with postcapillary venules, CTLs secrete a small amount of GzmB that works like scissors to cut through basement membrane constituents to facilitate their recruitment into the inflammatory site (32).

## 4 Clinical and therapeutic implications

Due to the distinct roles in various physiological and pathological contexts, GzmB emerges as a key contributor to immune regulation and disease pathogenesis. Initially recognized for its involvement in immune surveillance through cytotoxic lymphocyte-mediated apoptosis, GzmB has extended its influence into several realms, including autoimmune diseases, inflammatory disorders, neurological disorders, cancer immunotherapies, and allogeneic hematopoietic cell transplantation. Each area presents a unique interplay between GzmB and the intricate mechanisms governing immune responses and tissue homeostasis.

### 4.1 Autoimmune and inflammatory disorders

In an autoimmune condition such as rheumatoid arthritis, characterized by immune system attacking normal self-tissues, especially joints, GzmB may play a significant extracellular role, contributing to tissue damage (9). Its significance even extends to cardiovascular pathogenesis. Atherosclerosis, a major contributor to heart attacks and strokes, involves lipid-driven inflammation where T lymphocytes, macrophages, and other immune cells contribute to plaque formation. Mast cell-derived GzmB may induce detachment of endothelial and smooth muscle cells, implicating in atherosclerotic lesions (7). Elevated GzmB levels correlate with unstable plaques and post-infarct ventricular remodeling, signifying its potential role in disease progression. In arterial walls, inflammatory responses attract immune cells that secrete GzmB, which may contribute to arterial wall damage and susceptibility to aortic dilation and aneurysms. Two reports highlighted GzmB’s role in aneurysm formation independent of Perforin, positioning it as a potential therapeutic target (33, 34). Studies in murine models show that GzmB deficiency reduces aneurysm incidence, emphasizing its involvement in aneurysm progression. Elevated GzmB levels and the cleavage of fibrillin-1 signify its crucial role in vessel wall destabilization, a hallmark of aneurysm pathology. Further exploration of SA3N’s effects on GzmB and decorin degradation presents a promising avenue targeting GzmB for therapeutic intervention in aortic aneurysms. Furthermore, a few studies have implicated GzmB in T cell-mediated neurological disorders. GzmB released from CD4+ and CD8+ T cells can induce considerable neuronal injury. Recombinant GzmB, even at low concentrations in the absence of Perforin, induced significant toxicity in neurons through Giα-coupled receptors (11). GzmB’s interaction with neuronal receptors, particularly PAR-1 and Kv1.3, contributed to progression of neuronal damage (12). These findings not only shed light on the intricacies of GzmB’s role in neurotoxicity but also open promising therapeutic avenues for addressing T cell-mediated neurological diseases.

### 4.2 Cancer immunity, immune suppression and immunotherapy

Conventional treatments struggle against tumor progression, but emerging therapies including cancer vaccines and adoptive T cell therapy face challenges in generating effective immune responses due to immunosuppression. Innovative strategies in identifying tumor antigens, counteracting immune checkpoint and tumor-induced immune suppression have been fueling the growth of cancer immunotherapy. In this context, GzmB has exhibited complex roles in antitumor cytotoxic lymphocytes as well as immunosuppressive pDCs and Treg cells (9, 35, 36). Recent trials combining chemotherapy with immunotherapy show promise but still lack clarity in mechanisms. A study in 2010 highlighted chemotherapy’s role in enhancing tumor susceptibility to CTL-mediated killing. Chemotherapy, TAX, DOX, and CIS, made tumor cells more susceptible to GzmB-induced cell death independently of Perforin, facilitating non-antigen-specific CTL killing. This effect was mediated via upregulation of M6PR on the surface of tumor cells (37). These models suggest that combining radiation/chemotherapy with cancer vaccines enhances T cell responses associated with antitumor effects. Further studies are warranted to delineate the relevant mechanisms and design GzmB-targeting strategies that improve combinatory cancer treatments.

### 4.3 Allogenic hematopoietic cell transplantation

Many publications have provided strong evidence showing the significance of Perforin and GzmB in allogeneic hematopoietic cell transplantation (38). Several studies suggest that GzmB plays differential roles in graft-versus-host disease (GVHD) and graft-versus-tumor effect mediated by different types of donor T cells. While GzmB is required for CD8+ T cells to cause GVHD, GzmB-mediated damage of CD8+ T cells impairs graft-versus-tumor effect (4). On the other hand, GzmB-mediated activation-induced death of CD4+ T cells inhibits murine acute GVHD (39), while GzmB contributes to the optimal graft-versus-tumor effect mediated by CD4+ T cells (40). Furthermore, the endogenous inhibitor of GzmB, serine protease inhibitor 6, protects alloreactive T cells from GzmB-mediated mitochondrial damage without affecting graft-versus-tumor effect (41). Notably, host-derived serine protease inhibitor 6 also provides GzmB-independent protection of intestinal epithelial cells in acute GVHD (42), suggesting that it may interact with other proteases. These studies have underscored its complex roles in adverse GVHD and the desired graft-versus-tumor effect. There are ongoing studies investigating the roles of Perforin-independent GzmB activity in both acute and chronic GVHD models. Understanding the nuanced regulation and function of GzmB in allogeneic immune response may offer a promising avenue for managing GVHD severity.

## 5 Conclusion

In conclusion, the diverse roles of GzmB in cytotoxic and non-cytotoxic immune responses, its finely regulated gene expression, release by immune cells, and delivery to target cells through both Perforin-dependent and Perforin-independent mechanisms, underscore its significance in various physiological and pathological contexts. Its participation in extracellular and intracellular processes reflects dynamic versatility, while its dual mechanisms of delivery contribute to the complexity of immune responses. GzmB not only plays a critical role in immune surveillance and defense against pathogens, but also contributes to the regulation of immune homeostasis, mediating autoimmune responses and inflammatory processes. Therefore, the significance and versatility of GzmB call for researchers to delve deeper into the complexities of its involvement in inflammatory disorders, autoimmune diseases, and cancer. This evolving landscape holds great potential not only for enhancing our grasp of fundamental immunological processes but also for paving the way towards innovative interventions harnessing GzmB activities for targeted therapies in the treatment of a spectrum of medical conditions.

## Author contributions

RT: Writing – original draft. XC: Conceptualization, Writing – review & editing.
